# Osmotic demyelination syndrome with normal serum sodium in an alcohol-dependent patient: a case report

**DOI:** 10.3389/fnhum.2025.1688210

**Published:** 2025-11-27

**Authors:** Yaqi Li, Lu Han, Yumeng Jiang, Zhihong Zhao, Zikai Xin, Zilong Zhu

**Affiliations:** 1 Clinical College of Neurology, Neurosurgery and Neurorehabilitation, Tianjin Medical University, Tianjin, China; 2 Department of Electroencephalogram, Tianjin Huanhu Hospital, Tianjin, China; 3 Department of Neurology, Tianjin Huanhu Hospital, Tianjin, China

**Keywords:** osmotic demyelination syndrome, chronic alcoholism, case report, literature review, corticosteroid

## Abstract

Osmotic demyelination syndrome (ODS), which embraces central pontine myelinolysis and extrapontine myelinolysis, is a well-known but uncommon disorder of the central nervous system. The disease primarily occurs after rapid correction of severe hyponatremia. However, excessive drinking is one of the rare etiologies of ODS. Cases of alcohol-related ODS without documented hyponatremia are rarely reported, and optimal therapeutic approaches remain unclear. We report a patient in his 30’s with a history of heavy drinking who presented with unsteady gait and limb tremor as the main clinical manifestations. The patient consistently denied any history of fluid or electrolyte imbalances and reported a normal-range blood sodium level. Magnetic resonance imaging (MRI) revealed triangular T2-weighted and FLAIR pontine hyperintensity with a surrounding DWI rim. Finally, the patient recovered completely following corticosteroid treatment. This case shows the possibility that Alcohol-related ODS can occur without electrolyte disturbances and may respond favorably to combined corticosteroid and vitamin B therapy, warranting further investigation in clinical studies. We conducted a literature review of ODS in alcoholic patients and summarized its possible etiology, epidemiology, clinical characteristics and treatment options to raise awareness of such disorders.

## 1 Introduction

Osmotic demyelination syndrome (ODS), a rare neurological disorder characterized by demyelination of pontine white matter, is a subtype of osmotic demyelination syndrome. Although rapid sodium correction is the classical etiology (Danyalian and Heller, 2025), emerging evidence highlights chronic alcoholism as an independent risk factor. The pathomechanism of Alcohol-related ODS may involve some degree of electrolyte disturbance, as seen in dispotomania, as well as the role of ethanol in brain conduction distrubances (Giglio et al., 2024; Zahr and Pfefferbaum, 2017). Clinical manifestations of ODS range from dysarthria, dysphagia, and spastic quadriparesis to life-threatening locked-in syndrome (Sun et al., 2023). Magnetic resonance imaging (MRI) serves as the gold standard for diagnosis, typically revealing symmetrical pontine lesions. Extrapontine involvement, such as basal ganglia or cerebellar demyelination, may coexist (Alleman, 2014). Currently, there is no established treatment for ODS. Nutritional support, vitamin supplementation and immunomodulatory therapies have shown variable efficacy (Falhammar et al., 2025; Jamil et al., 2023; Nimodia et al., 2024; Tiwari and Kumari, 2022). Notably, Alcohol-related ODS may carry a better prognosis compared to electrolyte imbalance-associated cases. This report presents a case of ODS associated with Alcohol-induced that exhibited atypical imaging findings and achieved complete recovery following steroid hormone therapy. It is noteworthy that this patient has no history of hyponatremia nor its rapid correction. The report synthesizes existing literature to refine clinical understanding.

## 2 Case presentation

A male in his 30’s was admitted for progressive limb dyskinesia and bilateral hand tremors over 1 week. Initial symptoms included gait instability, forward-leaning posture, and mild tremors, which acutely worsened 2 days prior to admission, accompanied by lethargy. On hospital admission, patients’ vital signs were within the normal range. Physical examination reveals normal development and no evidence of dehydration. Neurological examination demonstrated mild somnolence with otherwise normal higher mental functions. The initial GCS was 14. Cranial nerve functions, including pupillary reflexes and extraocular movements, were intact. Motor examination revealed increased muscle tone, truncal ataxia, lower limb dysmetria, and Babinski sign was positive. During the medical history collection process, the patient consistently denied any history of fluid or electrolyte imbalances or medical interventions to correct such imbalances. As far as we know, no similar neurological symptoms have been reported among the patient’s immediate family members. The patient works as a truck driver, and his heavy workload has contributed to a prolonged history (>20 years) of excessive alcohol consumption (daily intake: ∼500 mL of distilled spirits) and smoking (20 cigarettes/day).

**FIGURE 1:**
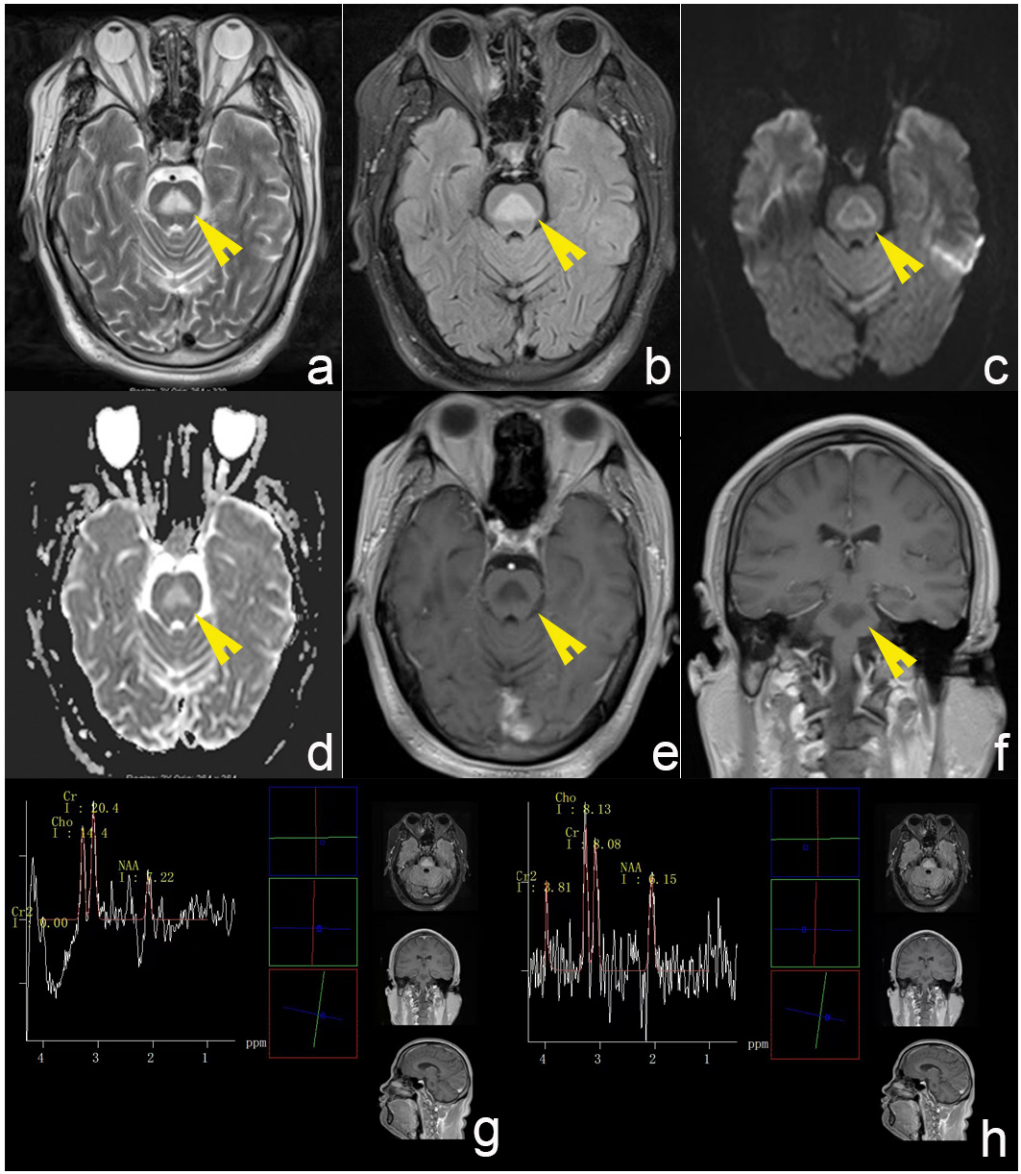
Brain MRI of the patient. **(a,b)** T2-weighted and FLAIR sequences revealed a triangular hyperintense signal within the central pons. **(c)** DWI revealed a markedly hyperintense rim surrounding the lesion. **(d)** ADC map showed corresponding hyperintensity in the lesion’s central region. **(e,f)** Post-contrast imaging revealed no areas of abnormal enhancement. The white arrows indicate lesion regions. **(g,h)** MRS indicated a significant decrease in N-acetylaspartate (NAA)/creatine (Cr) ratio and an increased choline (Cho)/Cr ratio. The yellow arrows denote diseased areas.

Laboratory findings were unremarkable except for elevated gamma-glutamyl transferase (167 U/L) and C-reactive protein (3.73 mg/L). His blood sodium level within the normal range (141 mmol/L). Cerebrospinal fluid analysis (CSF) ruled out infectious or inflammatory etiologies (protein: 0.41 g/L, leukocytes: 2 × 10^6/L; negative AQP4, MOG, MBP, GFAP antibodies and oligoclonal bands in plasma and CSF; negative high-throughput sequencing of pathogenic microorganisms). For the purpose of definite diagnosis, brain MRI was performed in the initial course of the disease. MRI revealed a triangular hyperintense signal within the central pons on T2-weighted and fluid-attenuated inversion recovery (FLAIR) sequences. Diffusion-weighted imaging (DWI) revealed a markedly hyperintense rim surrounding the lesion, while the apparent diffusion coefficient (ADC) map showed corresponding hyperintensity in the lesion’s central region. Post-contrast imaging revealed no areas of abnormal enhancement (Figure 1). Magnetic resonance spectroscopy (MRS) indicated a significant decrease in N-acetylaspartate (NAA)/creatine (Cr) ratio and an increased choline (Cho)/Cr ratio (Figure 1).

Initial diagnostic challenges included differentiation from Wernicke’s encephalopathy particularly for the absence of typical electrolyte disturbances. Notably, Wernicke’s encephalopathy exhibits characteristic MR imaging findings, with bilateral symmetric involvement being a common feature. It predominantly affects areas such as the mamillary bodies, thalamus, periaqueductal gray matter, hypothalamus, and tectum. Lesions in the mamillary bodies are particularly characteristic, presenting as T2/FLAIR hyperintensity. Contrast-enhanced scans may show enhancement in the mamillary bodies in some patients (Arendt et al., 2024). These manifestations exhibit significant distinctions from ODS. Furthermore, Wernicke’s encephalopathy is characterized by abnormal eye movements and nystagmus, typically accompanied by reduced serum thiamine levels. It demonstrates marked responsiveness to thiamine therapy, which serves as a key distinguishing feature from ODS (Hiraga et al., 2024). In our case, the typical clinical manifestations, laboratory findings, and imaging features of Wernicke’s encephalopathy as described above were not observed. Therefore, we excluded this diagnosis. In the absence of management guidelines for alcohol-related ODS, our decision to use glucocorticoid pulse therapy and vitamin was driven by the anti-inflammatory hypothesis and grounded in prior studies of ODS (Jamil et al., 2023; Nimodia et al., 2024; Tiwari and Kumari, 2022). The patient received intravenous methylprednisolone (1 g IV qd for 5 days) and vitamin B (100 mg im bid for 5 days). Gradual improvement in ataxia and tremors was observed. The patient was discharged on vitamin B oral therapy (10 mg po tid for 90 days). The patient did not report any treatment-related side effects. During the 3-months subsequent follow-up, the patient reported complete resolution of symptoms. Bilateral hand tremors, unsteady gait, and postural instability had fully resolved, and the patient had resumed his occupational activities. However, the patient declined further MRI reexamination. Consequently, it must be acknowledged that clinical diagnosis and treatment are constrained by patients’ limited understanding of ODS and low treatment compliance. The patient’s diagnosis and treatment timeline is shown in Table 1. We assessed the patient’s muscle tone [using the Modified Ashworth Scale (Harb et al., 2025)], ataxia and limb dysmetria [via the International Cooperative Ataxia Rating Scale (Perez-Lloret et al., 2021)], during hospitalization and follow-up, as detailed in Table 2.

**TABLE 1:** The patient’s diagnosis and treatment timeline.

Timeline	Diagnosis and treatment
1 week prior	Progressive limb dyskinesia, tremors, gait instability
2 days prior	Acute worsening with lethargy
Day 0	Hospital admission, initial Na^+^ 141 mmol/L
Day 5	Treatment initiation (methylprednisolone + vitamin B)
Day 7	First MRI showing ODS
Post-treatment	Gradual improvement
3 months	Complete symptom resolution

**TABLE 2:** The motor function assessment of the patient before and after treatment and follow-up.

Motor function assessment items	Before treatment	After treatment	Follow-up
Muscle tone (modified Ashworth Scale)	2	1	0
Truncal ataxia (International Cooperative Ataxia Rating Scale)	28	18	0

The case and imaging data presented above were obtained with written informed consent from the patient.

## 3 Discussion

Osmotic demyelination syndrome was first reported in 1959 and is commonly seen in the rapid correction of hyponatremia (Lambeck et al., 2019). Among adults, the overall prevalence of ODS was 0.4%–0.56% and even more uncommon in children (Bansal and Zinkus, 2019). Although the incidence is relatively low, its severe neurological impairment and potentially fatal nature make it a major concern in both neurology and internal medicine clinical practice. The core pathological feature of ODS is symmetrical demyelination, predominantly involving the central pons and other cerebral white matter tracts (Alleman, 2014). Classically, this condition is attributed to rapid correction of chronic hyponatremia, which leads to diminished osmotic adaptation in brain cells, subsequently causing cellular dehydration and myelin damage. Oligodendrocytes, being particularly vulnerable to volumetric changes, represent the primary target cells (Ambati et al., 2023; Yu and Wang, 2024). However, new evidence establishes that ODS can occur in patients without evidence of water and electrolytes disturbances. Such patients commonly have other risk factors, such as Alcohol abuse, antidepressant usage, malnutrition, diabetes or liver and kidney disease (Ambati et al., 2023; Erkalaycı et al., 2024; Jahan et al., 2020; Ormonde et al., 2020; Shrestha et al., 2023). The underlying mechanisms of how these factors cause ODS are still unclear. Some scholars thought that these conditions may cause glial dysfunction, apoptosis and fluctuate the extracellular osmotic environment, thereby increasing susceptibility to demyelination (Danışman et al., 2022; Erkalaycı et al., 2024; Kleinschmidt-Demasters et al., 2006; Shrestha et al., 2023). It is noteworthy that multiple risk factors can act synergistically to contribute to the development of ODS. As reported by Giglio et al. (2024), this synergy can lead to a spectrum of clinical features in ODS arising from different etiologies. According to previous study, approximately 27%–39% of ODS patients were found to have alcohol use disorder (Kleinschmidt-Demasters et al., 2006; Lv et al., 2021). In addition, among roughly 600 alcoholic patients examined with a 1.0-T MRI device, 11 were retrospectively identified as having a central pontine lesion, considered a sequela of ODS. These studies indicated chronic Alcohol consumption is one of the main etiologies of ODS. In chronic alcoholics, the uptake of neurotransmitters such as glutamate and GABA is inhibited, leading to excitotoxic injury. Alcohol also impairs glucose uptake by astrocytes and reduces the synthesis of both DNA and proteins. Alcohol suppresses the proliferation of oligodendrocyte precursor cells and decreases the expression of specific connexins, which play crucial roles in myelin maintenance (Ambati et al., 2023). These pathological mechanisms may partially account for the occurrence of demyelination in chronic Alcohol users with normal serum sodium levels in our case report.

Osmotic demyelination syndrome lesions primarily involve the myelin sheaths in the pons, resulting in central pontine myelinolysis (CPM) as the predominant manifestation. Nevertheless, demyelination may also occur in extrapontine myelin structures, such as the putamen, caudate nucleus, thalamus, lateral geniculate body, cerebral white matter, and peripheral cortical areas. This is often referred to as extrapontine myelinolysis (EPM) (Singh et al., 2014; Yu and Wang, 2024). The Mayo Clinic conducted a retrospective study of 23 ODS patients without etiological classification revealed CPM in 14 patients and EPM involvement in 10 patients (Graff-Radford et al., 2011). This suggests that EPS is not atypical in ODS. Interestingly, a retrospective study by Uchino et al. (2003) evaluated brain MRI findings in approximately 600 Alcohol-dependent patients, none of whom had a documented history of hyponatremia. The study identified the pontine lesion was located at the center of the basis pontis in all 11 patients. Additionally, bilateral symmetrical oval lesions were faintly visible in the middle cerebellar peduncles in 3 of the 11 patients. However, no patient showed symmetrical lesions in the basal ganglia or thalamus indicative of EPM (Uchino et al., 2003). Hence, we hypothesize various causes of ODS may differentially influence the sites and morphology of lesions. Central pontine myelinolysis may be more prevalent in Alcohol-related ODS. Nevertheless, a large sample size study is needed to confirm this conjecture in the future.

Clinically, ODS presents with a broad spectrum of symptoms, ranging from asymptomatic cases to severe manifestations such as coma or a vegetative state. The variability in clinical presentation is largely determined by the location of the demyelinating lesions (Lambeck et al., 2019; Singh et al., 2014). Lesions confined to the pons typically affect the corticospinal and corticobulbar tracts, resulting in motor dysfunction that manifests as dysphagia, dysarthria, and quadriplegia. When the demyelination extends beyond the pons, patients may exhibit a range of additional neurological deficits, including movement disorders, behavioral and cognitive impairments, epileptic seizures, polyneuropathy, or other peripheral neuropathies (Guo et al., 2023; Yu and Wang, 2024). Given the relative rarity of ODS, the majority of available reports are case reports or case series. Consequently, the factors underlying the severity of its clinical manifestations remain incompletely understood. Analysis of the literature on Alcohol-associated ODS reveals that affected patients typically manifest mild-to-moderate neurological disturbances and generally exhibit a favorable prognosis (An et al., 2010; Feng et al., 2016; Hagiwara et al., 2008; Ocak et al., 2020; Ozgur-Ilhan et al., 2005). This observation aligns with our case, in which the patient presented with mild symptoms, including ataxia and mild disturbances of consciousness. However, whether distinct etiologies are determinants of ODS severity requires confirmation through large-scale studies.

MRI serves as the gold standard for diagnosing ODS, characterized by symmetric T2-weighted and FLAIR hyperintensity in the central pontine base (Beh, 2017; Singh et al., 2014). Lesions typically concentrate in the central pons with relative sparing of peripheral pontine areas, classically presenting as the “trident sign” and “piglet sign” (Balcerac et al., 2021; Beh, 2017; Koul et al., 2023). Other lesion morphologies observed include occur, such as round, oval, linear, triangular, square, or star-shaped (Uchino et al., 2003). Singh et al. (2014) demonstrated that approximately 25% of patients with ODS manifested concomitant EPM, while 6.2% exhibited exclusively extrapontine involvement, most commonly affecting the basal ganglia, thalamus, cerebellum, and cerebral white matter. Similarly, these lesions typically demonstrate bilateral symmetric distribution (Aoki et al., 2016; Beh, 2017; Higinbotham and Nayate, 2022). Abnormal gadolinium enhancement was observed in only 20% of reported cases, typically peaking at lesion margins and occurring primarily in the early disease stages (Beh, 2017; Singh et al., 2014). DWI can detect abnormalities at the earliest stages, visible from 24 h to a week post-symptom onset, and it generally returns to normal in 3–4 weeks. Nevertheless, the specific manifestations of DWI abnormalities have not been described in detail (Beh, 2017; Ruzek et al., 2004). In addition to MRI, MRS can reveal alterations in myelin metabolism, aiding in the assessment of pathological processes in patients with ODS. MRS findings include abnormal myelin metabolism, characterized by decreased levels of common metabolites such as NAA, indicative of neuronal injury, while elevated Cho may be associated with gliosis (Guo et al., 2006; Shen et al., 2020). In our case, the patient exhibited symmetrically distributed T2 and FLAIR hyperintensity in the central pons without significant abnormal enhancement. MRS revealed a significantly decreased NAA/Cr ratio and a markedly elevated Cho/Cr ratio. These findings align with the well-established MR characteristics of ODS reported in the literature. Notably, MRI in this patient demonstrated triangular abnormal T2 and FLAIR signal shadow, a pattern infrequently documented in the literature (Kang et al., 2012; Uchino et al., 2003). However, the underlying mechanisms of these imaging findings and their potential impact on disease severity and prognostic outcomes remain unclear. Additionally, the peripheral hyperintensity observed on DWI has also been reported only in a minority of cases (Kang et al., 2012). We postulate that these atypical imaging features may correlate with the specific disease phase and mode of clinical onset. Future studies with larger sample sizes may be required to elucidate the clinical significance of these particular MRI findings.

Currently, there is no established treatment for ODS, making prevention crucial. The clinician should prevent the overcorrection of serum Na in the initial management of severe cases (Erkalaycı et al., 2024; Falhammar et al., 2025). Nutritional support plays a vital role in the recovery of patients suffering from central pontine myelinolysis, particularly those with chronic alcoholism. The supplementation of B vitamins, especially B1 (thiamine) and B12, is critical as deficiencies in these vitamins can lead to neurological complications. Studies have shown that adequate nutritional support can enhance recovery and improve neurological function in patients with ODS (Danışman et al., 2022; Jamil et al., 2023; Tiwari and Kumari, 2022). In recent years, Corticosteroids and immunomodulatory agents such as intravenous immunoglobulin (IVIG) have shown promise in managing the inflammatory responses associated with ODS (Nimodia et al., 2024). However, robust evidence to support this therapy has been lacking. In our case, short-term systemic corticosteroids and vitamin B complex led to rapid resolution of the patient’s symptoms, suggesting that it may be an effective therapeutic strategy for Alcohol-related ODS, it is speculated that this observation may be associated with the anti-inflammatory effects of steroid hormones. ODS demonstrates favorable recovery in over half of the cases, with mortality declining per decade. Even patients presenting with severe neurological manifestations may achieve a favorable prognosis (Singh et al., 2014). Lesion location, volume, diffusion restriction, and contrast enhancement appear to have no significant impact on prognosis (Beh, 2017). According to previous literature, Alcohol-related osmotic demyelination syndrome (ODS) may carry a generally favorable prognosis (An et al., 2010; Feng et al., 2016; Hagiwara et al., 2008; Ocak et al., 2020; Ozgur-Ilhan et al., 2005), whereas in liver transplant recipients, the prognosis may be less optimistic (Singh et al., 2014). We speculate that several factors may underlie this phenomenon. First, the toxic effects of long-term alcohol consumption on myelin cells appear to be more gradual and persistent than those associated with hyponatremia, and the body may possess a greater adaptive capacity to demyelination than to the rapid correction of hyponatremia. Second, corticosteroids, as lipophilic hormones, exert anti-inflammatory effects by regulating the expression of anti-inflammatory genes at the cellular level. In terms of their protective role against alcohol-induced toxicity, the anti-inflammatory action of corticosteroids may hold greater clinical relevance than the pathological consequences of osmotic demyelination syndrome (ODS) arising from osmotic imbalances (Cain and Cidlowski, 2017). Lastly, in this case, the patient was a young adult with no history of substance abuse or comorbidities, which likely contributed positively to the treatment outcome.

### 3.1 Limitations

This case report has several limitations that should be acknowledged. First, as a report of a single case, the generalizability of our findings is inherently limited. The presentation and outcomes may not be representative of all patients with similar conditions. Second, follow-up MRI was omitted due to the patient’s complete symptomatic recovery and subsequent refusal, in light of the fact that the result would not have changed the treatment plan. Finally, the nature of a case report precludes definitive establishment of causation between the administered treatment and the patient’s recovery. The favorable outcome may be attributed to the treatment, the natural history of the disease, or other confounding factors.

## 4 Conclusion

Osmotic demyelination syndrome is a rare demyelinating disorder. Etiological factors include rapid correction of chronic hyponatremia, chronic alcoholism, antidepressant use, malnutrition, diabetes mellitus, and hepatic or renal diseases. ODS lesions primarily affect pontine myelin but may also involve extrapontine myelin. Characteristic MRI findings demonstrate symmetrical T2 and FLAIR hyperintensity, typically without significant diffusion restriction or contrast enhancement. MRS facilitates diagnosis and differentiation from neoplastic etiologies. Short-term combined systemic corticosteroid and vitamin B complex therapy may represent a therapeutic strategy for Alcohol-related ODS. However, future large-scale clinical studies are warranted to clarify lesion characteristics, prognostic determinants, and optimal treatment regimens across different etiologies of ODS.

## Data Availability

The original contributions presented in this study are included in this article/Supplementary material, further inquiries can be directed to the corresponding author.
